# Spanking and Children’s Approaches to Learning: Estimates from a Longitudinal Matched-Sample Design

**DOI:** 10.3390/bs15050658

**Published:** 2025-05-12

**Authors:** Jeehye Kang, Christina M. Rodriguez

**Affiliations:** 1 Department of Sociology & Criminal Justice, Old Dominion University, Norfolk, VA 23529, USA; 2 Department of Psychology, Old Dominion University, Norfolk, VA 23529, USA; crodriguez@odu.edu

**Keywords:** approaches to learning, ECLS-K: 2011, entropy balancing, spanking, physical discipline

## Abstract

One form of corporal punishment commonly used in the United States is spanking. Spanking is a well-known risk factor for adverse child development, although its influence on children’s approaches to learning (ATL) has been largely overlooked. Existing research is particularly limited in inadequately considering multiple confounds and selection biases in children’s exposure to spanking. This study examined the links between spanking and children’s ATL, using a matched-group design to strengthen causal estimates among children aged 5 to 7.5 (N = ~12,800) from the Early Childhood Longitudinal Study, Kindergarten Class of 2010–2011. Entropy-balanced matching mitigated selection and confounding biases, controlling for a wide array of covariates. The sensitivity of spanking’s effects on ATL was also tested by limiting the sample to low-frequency spanking (once in the past week) to address concerns that primarily higher-frequency spanking predicts ATL. Findings indicated that spanking at age 5.5 was associated with less positive ATL at ages 6.5 and 7.5. These results remained significant when limited to low-frequency spanking. This study’s findings suggest that spanking may hinder children’s development of positive approaches to learning, which holds significant implications for lifelong well-being. This study contributes to the growing literature on the potential negative effects of physical punishment on child development across multiple domains.

## 1. Introduction

Spanking is a common and legally sanctioned disciplinary practice that is normative in the United States (Afifi et al., 2017). Defined as “the use of physical force with the intention of causing pain, but not injury, for the purpose of correcting or controlling the child’s behavior” (Donnelly & Straus, 2008, p. 3), spanking in the U.S. is considered to be non-abusive. Despite societal acceptance, spanking is a well-known risk factor for adverse child developmental outcomes (Avezum et al., 2023). Previous research has documented connections between spanking and children’s behavioral functioning (including problem behaviors; Avezum et al., 2023; Gershoff & Grogan-Kaylor, 2016), and recent studies have identified linkages to poorer cognitive outcomes (Kang, 2022a; Kang & Rodriguez, 2023; Tran et al., 2021; Wang et al., 2023). These findings suggest that the adverse effects of the common practice of spanking may be broad rather than concentrated in one domain of child development, pointing to the need to expand our understanding of the influence of parental spanking.

Often overlooked in previous research on spanking is its association with children’s approaches to learning—an important developmental outcome (Follari, 2018; Hyson, 2008; Puccioni, 2015). Approaches to learning (ATL) refers to learning-related emotions (e.g., eagerness to learn) and behaviors (e.g., paying attention and persistence in task completion), as well as self-regulated classroom skills (e.g., following classroom rules and organizing belongings; Hyson, 2008; Razza et al., 2015). Problematic ATL relates not only to academic functioning in school (Li-Grining et al., 2010; Razza et al., 2015), but to aggression (Mattison et al., 2018), less positive peer relations (Ramírez et al., 2024), and language and cognitive skills development (Qi et al., 2023), suggesting ATL functions as a critical element to support children’s individual long-term well-being. Although a previous study suggested that spanking could contribute to less adaptive ATL (Buek, 2019), the study focused on family structure and parental involvement but did not consider confounding and selection biases associated with children’s exposure to spanking, which can increase the risk of inaccurately estimating its effects (Gershoff et al., 2018a; Larzelere et al., 2018). This study fills this gap by examining the association between spanking and later ATL using a matched-group design to mitigate confounds and selection biases. Given ATL’s development in early childhood and its stability (Kulkarni & Sullivan, 2022; Wu, 2022), understanding whether early spanking impacts children’s ATL can provide insight into processes that can affect a wide range of long-term indices of well-being.

### 1.1. Development of Approaches to Learning: Theoretical Issues

The development of children’s attitudes and learning behaviors has historically been viewed as a function of socialization processes, in which socially shared activities and behaviors are internalized (Vygotsky, 1978; Wertsch, 1991). In this process, social interaction is an essential element. Through social interactions, particularly with more experienced members of a community, such as parents, children acquire and develop the skills and functions needed to become competent members of the community (Vygotsky, 1978). Within this social constructivist approach to cognitive development, children’s ATL development is shaped by their social environment and the interactions they experience.

Family serves as the primary social context for children, central to early ATL development. From the perspectives of both attachment and self-determination theories, parents can condition and cultivate children’s motivations, attitudes, and behaviors for learning (Dweck, 2006). Attachment theory emphasizes the importance of primary attachment relationships in shaping human motivation and behavior. Given the central and salient nature of the parent-child relationship, children’s attachment to their parents influences their motivation and desire to explore their environment (Bowlby, 2004). Children with a secure attachment to their mothers tend to exhibit higher levels of engagement in and exploration of tasks (McCormick et al., 2016), as well as greater self-efficacy and confidence when facing new, challenging situations (A. Bender & Ingram, 2018; Haji Vosoogh et al., 2022). Based on attachment theory, this confidence and motivation to explore, engage in, and adapt to new environments is foundational to children’s positive ATL.

Self-determination theory emphasizes the critical role of parents in cultivating motivation in children. This theory rests on the assumption that humans have innate needs and desires to initiate actions of their own volition (i.e., autonomy), to master their environment in pursuit of desired goals (i.e., competence), and to experience closeness, connectedness, and belongingness with others (i.e., relatedness; Deci & Ryan, 2008). The development of these drives depends on the extent to which parents nurture these needs. Specifically, supporting children’s needs for autonomy, competence, and relatedness can enhance their intrinsic motivation, whereas controlling children’s achievement outcomes through extrinsic rewards and punishment may undermine their motivation (Ryan & Deci, 2020). Indeed, children whose views were considered, and who were allowed to make a choice with parental assistance, tended to have higher levels of teacher-rated self-motivation and interest in learning (Grolnick & Ryan, 1989). Thus, supporting children’s self-determination can lead to positive ATL (Ucar et al., 2024).

Aligned with the principles of both attachment and self-determination theories, positive parenting practices appear to foster children’s optimal ATL development. Positive parenting involves consistency, firm guidance, and non-violent practices characterized by warmth, sensitivity, nurturance, and acknowledgment of a child’s individuality, all of which encourage the child to reflect on and regulate their behavior (González-Cámara et al., 2019; Rajyaguru et al., 2019). Positive parenting practices, encompassing cognitive stimulation and emotional support, facilitate children’s self-regulated learning by strengthening their attachment security and increasing their motivation to achieve competence, thereby linking these practices to both attachment and self-determination theories (Bodovski & Youn, 2010; Gunderson et al., 2018; Kwok et al., 2023; Pino-Pasternak & Whitebread, 2010). Although the capacity for positive parenting can be constrained by limited family resources (Azad et al., 2014), previous studies have collectively conveyed that positive parenting approaches can shape the development of children’s adaptive ATL (e.g., Hill & Palacios, 2021; Kwok et al., 2023; J. Zhao et al., 2023).

### 1.2. Spanking and Children’s Approaches to Learning

Alternatively, from the perspectives of attachment and self-determination theories, spanking can impair children’s ATL. According to attachment theory, spanking disrupts children’s attachment security with their parents (Bowlby, 2004). Despite being intended as an educational corrective for misbehavior, spanking inflicts pain, which can confuse children because this parental action contradicts children’s expectations of nurturance and protection. Consequently, spanking can impede the development of secure attachment between children and their parents (Bowlby, 2004). Consistent with attachment theory, parents of insecurely attached preschoolers are more likely to have experienced corporal punishment (Cuartas, 2023). Children with insecure attachments also exhibited less noncompliance in a laboratory-based game situation; although those children may appear to be well-regulated, their behavior could reflect fear of being seen as difficult, demanding, or challenging in the eyes of their parents (Barnett et al., 1998). This fear, driven by the anticipation of punishment, may compromise their internalized motivation to autonomously explore and learn, undermining their self-determination. Indeed, children’s insecure attachment has been associated with lower task engagement in middle childhood (McCormick et al., 2016).

Further from the perspective of self-determination theory, spanking can hinder ATL development by undermining children’s sense of competence, autonomy, and relatedness (Holland & Ohle, 2020; Ryan & Deci, 2020). Positive ATL requires children’s optimism and confidence in their abilities (i.e., self-competence; Nurttila et al., 2015), willingness to make choices (i.e., autonomy; Alley, 2019), and interest in others’ thoughts and feelings (i.e., relatedness; Furrer & Skinner, 2003). However, spanking can undermine a sense of competence by focusing on what the child did wrong through negative feedback; spanking can weaken their sense of autonomy by using external forces to enforce compliance and by depriving opportunities for self-direction; and spanking can reduce a sense of relatedness by disregarding children’s emotions and failing to model warmth (H. L. Bender et al., 2007; Ryan & Deci, 2017). Used as a form of negative external feedback in response to children’s low academic performance, spanking can also lessen children’s intrinsic motivation to learn (Gottfried et al., 2009). Therefore, empirical evidence suggests that spanking can undermine children’s self-determination and secure attachment which in turn could compromise their ATL.

### 1.3. Spanking as a Risk Factor

Although negative parenting and punishment, in general, may weaken children’s positive ATL, the early onset and prevalence of spanking, along with growing evidence of its neurobiological consequences, imply that spanking is a specific risk factor. Spanking induces both physiological and emotional reactions, including stress and fear. Although the mechanisms by which these physiological effects interfere with children’s ability to engage in learning behaviors are unclear, corporal punishment has been linked to heightened neural stress responses (Cuartas et al., 2021) and neural fear responses (Knopf, 2021), similar to the effects observed in cases of physical abuse (Teicher et al., 2016; Tomoda et al., 2009), countering claims that spanking is harmless. Furthermore, parental use of spanking often begins early in a child’s life, driven by the popular but erroneous belief that spanking prevents more severe problems later on (Holden, 2020; Gershoff & Grogan-Kaylor, 2016). Despite recent declines (Mehus & Patrick, 2021), spanking remains prevalent. In the U.S., spanking can begin as early as 3 months of age (Zolotor et al., 2011), with at least 49% of parents self-reporting that they have spanked their children ages 0–9 (Finkelhor et al., 2019)–but self-reports may be underestimates. Although evidence documents the ineffectiveness and harms of spanking in the long term (Gershoff & Grogan-Kaylor, 2016), about 56% of American adults agree that it is sometimes necessary to spank a child (NORC at the University of Chicago, 2022). In sum, given its early onset, prevalence, and potential neurobiological consequences, spanking appears to confer specific developmental risks, with its relation to ATL being particularly relevant to study for young children.

### 1.4. Empirical Evidence Linking Spanking and ATL

A limited number of empirical studies imply possible linkages between spanking and children’s ATL. Using a longitudinal sample of U.S. children collected in 2011 (ECLS-K: 2011), parental use of spanking in kindergarten was negatively associated with children’s ATL in the 2nd grade, even after controlling for family SES, parental educational expectations (e.g., how far the parent foresaw their child achieving), and parental involvement in school activities (e.g., attendance at Parent Teacher Association meetings) (Buek, 2019). In a sample of Ghanaian preschoolers collected between 2015 and 2016, parental use of physical punishment predicted children’s lower ATL levels, whereas nonviolent punishment predicted higher ATL levels, even after controlling for family SES, parental marital status, and children’s socio-emotional skills (S. Wolf & Suntheimer, 2020); however, because this latter study’s measurement of physical punishment included some harsh practices like “beating [the child] up”, (S. Wolf & Suntheimer, 2020, p. 4) their findings should be interpreted with caution because their effects could be confounded by inclusion of high severity physical punishment or abuse. On the other hand, in a sample of U.S. children collected in 1998–1999 (ECLS-K), no association was observed between physical punishment in kindergarten and ATL in 5th grade (Bodovski & Youn, 2010); however, this study measured physical punishment combining elements of a coercive parenting environment (e.g., whether the parent would spank or hit back if the child hit them) with the frequency of spanking in the past week, and thus their null finding may not accurately reflect the isolated effects of parental spanking. In sum, the empirical evidence from one study that isolated the effects of spanking supports a negative link between spanking and ATL (Buek, 2019).

Although existing studies have used longitudinal samples with a small selection of covariates in their models (Buek, 2019; S. Wolf & Suntheimer, 2020), other covariates have not been properly controlled. For instance, children’s executive functioning (EF) skills have not been statistically controlled. EF skills are involved in regulating and organizing cognition and action required for learning and adaptation (Zelazo, 2020), often manifesting in their ability to override prepotent responses (i.e., inhibitory control), shift between tasks or mental sets (i.e., cognitive flexibility), and temporarily hold and manipulate information (i.e., working memory). EF skills relate to both ATL and exposure to spanking, as children’s stronger EF skills significantly predict better ATL (Vitiello & Greenfield, 2017), and children with lower EF skills are more likely to be spanked (Kang & Rodriguez, 2023). However, no previous studies on parental physical discipline and children’s ATL have controlled for children’s existing EFs. In addition, home environment factors have not been comprehensively controlled in previous research. Although some researchers have considered ATL in relation to cognitive stimulation at home (Buek, 2019), and others have controlled for emotional support (Bodovski & Youn, 2010), comprehensive controls for multiple aspects of the home environment, simultaneously with child and parent demographic factors, are rare in previous research.

### 1.5. Confounding and Selection Bias

A persistent challenge in empirically estimating the effects of spanking has been addressing confounds and selection biases as children cannot ethically be randomly assigned to experience spanking in experimental designs (see Gershoff et al., 2018a; Larzelere et al., 2018), yet families who spank may differ on multiple dimensions from those families who do not, including child and parent characteristics. For example, boys, children with externalizing behavioral problems, and children with disabilities (Bizzego et al., 2020; Chung et al., 2022) are all more likely to be spanked. Primary caregivers’ age (younger), race (Black), educational status (less than college), marital status (not married), poverty status (e.g., less than 100% poverty line), religiosity, depressive symptoms, parenting stress, and geographic locations (rural, Southern states) in the U.S., are significant predictors of spanking use as a disciplinary method (Chung et al., 2022; Gershoff et al., 2018b; S. J. Lee & Altschul, 2015; MacKenzie et al., 2011; Ward et al., 2021). Parents who spank also tend to show lower levels of parental warmth and cognitively stimulating activities (Gershoff et al., 2018b; Kang & Rodriguez, 2023). Notably, some of these characteristics (e.g., children’s EF skills or externalizing behaviors, low parental education) not only increase the likelihood of spanking but also relate to children’s ATL development (Buek, 2019; MacKenzie et al., 2015), confounding the estimates of the effects of spanking on children’s ATL. Additional factors related to ATL could affect estimates of the effects of spanking, such as children’s experience of center-based care before kindergarten (Fukkink et al., 2024), parental educational expectations (Pinquart & Ebeling, 2020), parental involvement in school activities (Anthony & Ogg, 2019), and type of school (e.g., public vs. private; Peterson & Llaudet, 2006; P. J. Wolf & Egalite, 2018).

Existing studies often attempt to address these selection biases by including potential confounds as covariates, but this approach is limited in most research designs. Non-random selection—both into who practices spanking (i.e., parental characteristics) and who experiences spanking (i.e., child characteristics)—remains a challenge, particularly in studies using simple model specifications. Simple model specifications, where estimates depend heavily on a researcher’s modeling choices, covariates, and measures of the key independent variable, can produce substantial variations in results, compromising the reliability of estimates (Ho et al., 2007). This issue becomes particularly concerning when the independent variable itself is confounded with the covariates. Failure to adequately address confounding and selection biases may affect the estimates of spanking’s effect on child outcomes (Larzelere et al., 2018).

In addition, concerns have been raised about the effects of spanking being conflated with those of potentially “excessive” spanking (Gershoff, 2016, p. 6), biasing the estimates of the effects of spanking. Excessive spanking can be an indicator of possible physical abuse (Gershoff, 2016; Gershoff et al., 2018a) and some have suggested that mild and/or infrequent spanking should be distinguished from excessive and/or frequent spanking when considering the potential effects on outcomes (Larzelere et al., 2010; Larzelere et al., 2017). Otherwise, the negative effects of frequent spanking may overgeneralize the harm of spanking that is infrequent. However, no previous research on parental spanking and children’s ATL has addressed this type of concern.

### 1.6. Current Study

The current study examined the association between spanking and later ATL, using a longitudinal matched-group design to mitigate confounds and selection biases. Matching is more effective than simple model specification, as it accounts for some of the limitations arising from non-random differences between spanked children (i.e., exposed group) and not-spanked children (i.e., unexposed comparison group; Cuartas et al., 2020; Gershoff et al., 2018b; Kang, 2022a, 2022b; Kang & Rodriguez, 2023; Okuzono et al., 2017). By preprocessing the data so that these groups are ‘matched’ in terms of a wide range of observed characteristics, this approach approximates the qualities of an experimental design (Ho et al., 2007; Stuart, 2010). The factors that simultaneously affect the likelihood of children being spanked and the development of ATL, as well as covariates that influence children’s ATL, were statistically controlled in matched analytical models. Additionally, to reduce bias associated with conflating any spanking with frequent spanking, the current study performed a sensitivity check of the main analysis by limiting the sample to infrequent spanking (i.e., spanked only once the prior week).

Drawing on the literature suggesting a link between physical punishment and children’s ATL (Buek, 2019; S. Wolf & Suntheimer, 2020), several hypotheses were proposed. First, spanking at age 5.5 years was expected to be associated with less positive ATL at age 6.5 years in matched samples. Second, spanking at age 5.5 years was predicted to be associated with less positive ATL at age 7.5 years in matched samples. Finally, extending the first two hypotheses, the negative association between spanking and children’s later ATL outcomes was predicted to be statistically significant even in cases of infrequent spanking.

## 2. Methods

### 2.1. Participants

This study drew a sample from the Early Childhood Longitudinal Study, Kindergarten Class of 2010–2011 (ECLS-K: 2011), the data collected by the U.S. Department of Education’s National Center for Educational Statistics (NCES). The ECLS-K: 2011 tracked a nationally representative sample of children from their kindergarten enrollment in the Fall of 2010/2011 through elementary school. A multistage probability sampling design was employed in which geographical areas were first selected and then schools within those areas for sampling children. Sample weights were applied to adjust for unequal selection probabilities and nonresponse effects, allowing nationally representative estimates to be generated (Tourangeau et al., 2015). In its base year, data were collected from 18,174 children, as well as their parents and teachers, across 968 schools nationwide. Response rates range from 74.3% to 79.8% for parents and from 79.5% to 86.8% for teachers, varying over the years. Sample weighting, however, minimizes concerns regarding nonresponse bias (Tourangeau et al., 2017). Notably, this study used restricted-use data, but public-use data files and user manuals are available online at https://nces.ed.gov/ecls/dataproducts.asp (accessed on 6 May 2025).

The present study utilized data from the first four waves of the ECLS-K: 2011, encompassing the fall (at age 5) and spring of kindergarten (at age 5.5), the spring of first grade (at age 6.5), and the spring of second grade (at age 7.5). To isolate the effects of parental spanking, about 630 children with non-biological mothers and/or fathers, as well as about 4650 cases missing information about the relationship with the “parent” respondent were excluded. The analytical sample consisted of about 12,800 children, rounded to the nearest 10, in accordance with NCES protocol. To account for missingness at item levels, ranging from 0–23% across variables, multiple imputation was employed to reduce bias and enhance efficiency (J. H. Lee & Huber, 2021; Madley-Dowd et al., 2019). The number of imputations (‘m’) was determined as recommended by White et al. (2011, p. 388), so that the fraction of missing information (FMI) divided by m should be approximately 0.01. In this study, 35 datasets were imputed, and the estimates derived from these imputations were integrated using Rubin’s rules (Carpenter & Smuk, 2021).

### 2.2. Measures

#### 2.2.1. Dependent Variables

The Approaches to Learning scale (ATL) was completed by teachers of children’s learning-related behaviors using the following items: keeping belongings organized, showing eagerness to learn new things, working independently, easily adapting to changes in routine, persisting in completing tasks, paying attention well, and following classroom rules. The seventh item (i.e., following classroom rules) was added to the first-grade wave. These items were developed specifically for the ECLS-K and were included in the Social Skills Rating System measure (Gresham & Elliott, 1990; Gresham et al., 2011). Teachers indicated the frequency of occurrence, with 1 indicating never, 2 indicating sometimes, 3 indicating often, and 4 indicating very often. The ATL scale score is the mean rating on the seven items (six items for age 5) included in the scale. ATL has an internal consistency estimate of 0.91 for each round of data collection (Tourangeau et al., 2015). The lagged dependent variables in the analysis were ATL measured in the spring of first grade (age 6.5) and in the spring of second grade (age 7.5).

#### 2.2.2. Independent Variable

In the spring of kindergarten, primary caregivers reported how often they spanked their children in the past week. Based on their responses, a binary variable was constructed: children who had not been spanked in the past week (0 = not spanked), and those who had been spanked at least once in the past week (1 = spanked). For the purposes of the analysis for the sensitivity check, children who had been spanked only once (excluding 2 or more times in the past week) were additionally identified. The spanking variable was dichotomized in order to create “exposed” and “unexposed” groups for the matching.

#### 2.2.3. Covariates Used in Matching and as Control Variables

Matching procedures incorporated a range of covariates including child, family, and geographical background characteristics. Child-level variables included: gender (coded as 0 = girl, 1 = boy); disability status (binary); age in months; prior experience of center-based early care and educational settings, such as daycare center, preschool, and prekindergarten (binary). Additionally, children’s baseline scores of ATL, externalizing behaviors, and executive functioning (EF) skills—assessed in the fall of kindergarten (at age 5)—were included as controls. Children’s baseline externalizing behaviors were measured by teachers’ ratings with five items on how often the children acted out (e.g., fighting, arguing, anger, impulsivity) or demonstrated disruptive classroom behaviors (α = 0.88) on a four-point Likert Scale. Children’s EF skills in the ECLS-K: 2011 were composed of children’s inhibitory control, cognitive flexibility, and working memory. The children’s inhibitory control score (α = 0.87) was measured through teacher report using 6 items (e.g., the ability to restrain or suppress behavior as needed in specific situations over the past six months; Putnam & Rothbart, 2006). The Dimensional Change Card Sort (DCCS) score was used to assess children’s cognitive flexibility (Zelazo, 2006; Zelazo et al., 2013), demonstrating strong test–retest reliabilities (α = 0.90 to 0.94; Beck et al., 2011; Zelazo et al., 2013). The Numbers Reversed subtest of the Woodcock-Johnson III (WJ III), which was used to assess working memory (Woodcock et al., 2001), has high reliability (about α = 0.88, Floyd et al., 2009); the W score, standardized score with a mean of 500 and standard deviation of 100, was employed because of its utility in tracking growth over time (Mather et al., 2001).

Parental demographic and behavioral characteristics included: primary caregivers’ race (non-Hispanic white, non-Hispanic black, Hispanic, Asian, other race); age; education level (<high school, high school, some college, bachelor’s degree); marital status (married or in a civil union, never married, previously married [separated, divorced, or widowed]); parenting stress; parental depressive symptoms; and engagement in child’s education. Parenting stress (α = 0.60) was assessed using the sum of 4 items rated on a four-point Likert scale (“Being a parent is hard”; “child does things that bother me”; “I sacrifice to meet the child’s needs”; and “I feel angry with the child”). Parental depressive symptoms were measured based on self-reports regarding the frequency of feeling depressed during the past week (1 = never, 2 = some of the time, 3 = a moderate amount of the time, 4 = most of the time). Educational engagement was measured with parental expectations for their children’s educational achievement (0 = less than college degree, 1 = college degree and higher), their participation in parent-teacher association (PTA) meetings (0 = no, 1 = yes), and their volunteering at the school since the start of the school year (0 = no, 1 = yes). About 99% of primary caregivers were mothers.

Home environment covariates included level of cognitive stimulation; emotional support; family religious beliefs or traditions; and family poverty status. Cognitive stimulation was assessed using a set of 9 items (e.g., telling stories, singing songs, helping children with art, playing games, discussing nature, building things, engaging in sports, practicing reading and writing numbers, and reading children books) on a four-point Likert scale (1 = not at all, 2 = once or twice a week, 3 = three to six times a week, and 4 = every day), and the sum of these items was calculated (α = 0.73). Emotional support (α = 0.67) was assessed by summing 4 items rated on a four-point Likert scale (1 = not at all true, 2 = somewhat true, 3 = mostly true, and 4 = completely true) to the following items: “having a warm and close time together”; “child likes me”; “showing the child love”; and “expressing affection.” Family religious beliefs or traditions were measured by the frequency of discussing religious beliefs or traditions at home (1 = never, 2 = almost never, 3 = several times a year, 4 = several times a month, 5 = several times a week or more). Family poverty status was categorized according to the primary caregivers’ reports of household income, with three levels: below the poverty line, 100–199% of the poverty line, 200% at or above the poverty line. Finally, geographic characteristics include U.S. regions (Northeast, Midwest, South, West) and urbanicity (city, suburb, town/rural).

### 2.3. Analytical Strategies

The analyses proceeded in two phases. In the first phase, matching was applied to 44 observed covariates across the 35 imputed samples, employing entropy balancing. Entropy balancing (EB) is a matching technique that re-weights observations such that the specified moments (e.g., mean, variance) of the covariate distributions of the weighted unexposed comparison sample ‘match’ those of the exposed sample (Hainmueller, 2012). Compared with a propensity score matching approach which estimates a distance metric (e.g., a difference in propensity scores) for matching, EB’s direct incorporation of covariate balance into the weight function always (however weakly) ensures covariate balance (Hainmueller, 2012; Hainmueller & Xu, 2013). This direct method prevents the issue seen in propensity score matching, where improving balance on some covariates may worsen it on others (Iacus et al., 2012). In the current study, a set of entropy-balanced samples based on the incidence of spanking was generated to examine the associations with ATL.

In the second phase, lagged dependent variable (LDV) regression analyses were conducted on the multiple imputed and entropy balanced samples. The ATL outcomes at ages 6.5 and 7.5, respectively, were regressed on the spanking exposure variables. To further reduce confounding errors, regression models included all the covariates and baseline ATL (at age 5) that preceded spanking report at age 5.5 that were used in the matching model (Q. Zhao & Percival, 2017). Finally, for the sensitivity check, the models were replicated by limiting the sample to cases where children who had not been spanked were compared with those who had reportedly been spanked only once in the previous week (i.e., not spanked versus spanked once).

## 3. Results

### 3.1. Preliminary Results

Results show that about 83.5% of children were not spanked in the past week, and about 16.5% were reportedly spanked at least once in the past week. Specifically, about 10% had been spanked only once, and about 6.6% spanked two times or more.

Table 1 presents a comparison of the differences between the unmatched and matched samples based on spanking experiences (not-spanked vs. spanked). Notably, the sample sizes varied due to the variability across the imputed data sets—especially because the imputed children’s spanking experiences varied. Before matching, there were differences between the not-spanked and spanked groups on approximately 86% of the covariates. Specifically, spanked children were more likely to be boys, with disabilities, attending private school, and showed higher levels of externalizing behaviors, lower levels of EFs, and less positive ATL. Their primary caregivers were younger, more likely to be Black, Hispanic, or other Race, less likely to be married, more likely to show depressive symptoms, and more likely to report higher levels of parenting stress. Although parents had lower levels of educational expectations for their children and were less likely to volunteer at the school, there was no difference in attendance at PTA meetings. Those families using spanking for discipline showed lower levels of cognitive stimulation and emotional support, and higher levels of family poverty, and more engagement in family religious beliefs or traditions. Those using spanking were more likely to live in the South and in rural areas. After EB matching, the distributions of covariates were nearly identical between spanked and not-spanked children. Hence, entropy balancing successfully achieved a balance between the exposed and unexposed comparison groups on the covariates. Notably, the sample sizes after matching are larger compared to those before matching because the former utilized all cases whereas the latter were weighted using a sampling weight provided by NCES, which contained some “0” values.

### 3.2. Analytical Results

Table 2 displays the results of the LDV regression analyses with the unmatched and EB-matched samples’ ATL outcomes at ages 6.5 and 7.5. The estimates for spanking using the matched sub-sample excluding the more frequent spanking cases (i.e., limited to 0 vs. a single spanking) are also displayed.

Before EB-matching, those who were spanked at the age of 5.5 scored 0.079 points lower ATL at age 6.5 (*p* < 0.01) and 0.071 points lower ATL at age 7.5 (*p* < 0.05), compared with those who were not spanked. There were several statistically significant associations with parenting and home environment covariates. Primary caregivers’ greater parenting stress was associated with 0.033 points lower ATL at age 7.5. Family poverty status 200% above the poverty line (highest income level) was linked to children’s higher ATL, by 0.114 points at age 6.5 and 0.097 points at age 7.5, compared with families below the poverty line. Positive parenting practices, such as cognitive stimulation and emotional support, were not statistically significant (see Appendix A for specifics).

After EB-matching, spanking at age 5.5 was significantly associated with 0.081 points lower ATL at age 6.5 (*p* < 0.001) and 0.067 points lower ATL at age 7.5 (*p* < 0.01). Notably, both magnitude and statistical significance of the coefficient at age 6.5 increased after matching, whereas the magnitude of the coefficient at age 7.5 decreased but remained statistically significant. Parenting and home environment covariates showed similar patterns to those observed in unmatched samples, but the coefficient for the family poverty status between 100–199% of the poverty line, compared to those below the poverty line, increased in both magnitude and statistical significance for children’s ATL at age 7.5 (0.106, *p* < 0.01). This indicates some confounding between family poverty status and spanking in the unmatched data. The improved balance of covariates between exposure and control groups after matching reduced confounding errors, allowing more precise estimates for both the effects of spanking and the contributions of the covariates.

For the sensitivity check, the matching and analytical models were replicated, limiting the sample to 0 vs. only 1 spanking in the prior week. Spanking even once in the prior week at age 5.5 was associated with 0.071 lower ATL at age 6.5 (*p* < 0.05) and 0.070 points lower ATL at age 7.5 (*p* < 0.05). Between the full and subsamples, the magnitude of the coefficients at age 6.5 decreased by about 12% (from −0.081 to −0.071), whereas the coefficients for age 7.5 slightly increased by about 4.5% (from −0.067 to −0.070). All coefficients for spanking remained significant. The robustness of the findings supports that even infrequent spanking was linked to lower ATL scores. Parenting and home environment covariates exhibited similar patterns to those observed in the full matched samples, although their coefficients slightly reduced in magnitude (2–16%) possibly due to the smaller sample sizes.

## 4. Discussion

This study examined the link between children’s earlier spanking experience and their subsequent approaches to learning in school. Using lagged dependent variables to add temporality, and a matching design to mitigate confounding and selection biases in a large, nationally representative sample of US children, the current study better approximates causal estimates of the effects of spanking on children’s ATL relative to existing studies. In our matching design, a total of 44 theoretically and empirically relevant covariates were selected, including children’s cognitive and behavioral skills prior to spanking exposure at age 5.5, as well as parenting behaviors, and home environment. The study also ruled out confounds associated with frequent spanking by conducting a sensitivity check that analyzed the effects for a single instance of spanking. The findings support the hypotheses, indicating a significant connection between earlier spanking at age 5.5 and less positive ATL at ages 6.5 (Hypothesis 1) and 7.5 (Hypothesis 2), holding true even with infrequent spanking (Hypothesis 3). The use of a rigorous matching design, lagged dependent variables, and robustness check with infrequent spanking, not only improved upon existing models—which often fail to address confounding and selection biases—but also strengthened the potential causal interpretations between spanking experience and later ATL development. These results are consistent with the findings from previous (yet less statistically controlled) research (Buek, 2019), confirming the developmental risks of spanking for children’s ATL development. The harm posed by spanking, considered a non-abusive form of physical punishment, suggests that it is not a harmless practice and adds to the growing evidence of the adverse effects of spanking on child development across multiple domains.

The observed significant links between spanking experiences and ATL are consistent with attachment (Bowlby, 2004) and self-determination theories (Holland & Ohle, 2020; Ryan & Deci, 2020). Although examining each theoretical pathway in detail is beyond the scope of this study, spanking may disrupt the formation of secure attachment between parents and children, impeding children’s active engagement in exploring and adjusting to their surrounding environment (Barnett et al., 1998; McCormick et al., 2016; National Research Council & Institute of Medicine, 2000). From this perspective of attachment theory, this disruption can hinder children’s engagement in new learning environments. Additionally, as a form of external punishment, spanking may have undermined children’s sense of competence, autonomy, and relatedness—key psychological needs that are foundational for intrinsic motivation and engagement in learning (H. L. Bender et al., 2007; Ryan & Deci, 2017). From the perspective of self-determination theory, the resulting lack of positive feedback, opportunities for self-direction, and emotional resources may explain why spanked children show less positive ATL (Alley, 2019; Furrer & Skinner, 2003; Nurttila et al., 2015). The theoretical and empirical connections between spanking and adverse child development further support causal connections between spanking and ATL. Examining the mediating pathways that disentangle the mechanisms for how spanking impairs ATL would be a fruitful avenue for future research.

We found no significant associations between positive parenting practices such as cognitive stimulation and emotional support in relation to children’s ATL. In contrast, family stressors maintained a significant role in the development of children’s ATL. Family poverty status was associated with children’s ATL, with the larger disparity observed between families below the poverty line and those 200% above the poverty line—consistent with earlier work documenting that family poverty poses as a significant risk for children’s poor ATL development (Buek, 2019; Duncan et al., 2015). Greater parenting stress experienced by primary caregivers was also negatively associated with ATL, with the difference most pronounced at age 7.5. Although the null finding of positive parenting practices can be due to unobserved factors overshadowing or moderating their influence, the highly controlled models used in the study, which included 44 covariates, may also have dampened the beneficial influence of positive parenting. Given the effects of stressors (e.g., spanking, parenting stress, and poverty) emerging as significant in the same model, positive parenting might not be sufficient to mitigate these risks. This possibility about the limits of positive parenting on ATL warrants future research.

Children’s approaches to learning are important yet overlooked elements to child development. Positive ATL forms the bedrock upon which social, linguistic, and cognitive development are built (Mattison et al., 2018; Ramírez et al., 2024; Qi et al., 2023) to apply in a variety of environments. Children with an open, motivated mindset with an engaging attitude are likely to learn, apply, and retain knowledge, as well as to develop peer relationships through positive communication. The negative association between spanking and ATL suggests that this practice may undermine children’s capacity to thrive both socially and academically, with long-term implications. Given the early onset and prevalence of spanking, as well as the neural plasticity of early brain development (Cuartas et al., 2020), interventions targeting parenting practices in early childhood are likely to be meaningful. Parents would be best served to refrain from using corporal punishment if their parenting goal is raising children to express and exercise their desire to learn, work independently, persist in completing their tasks, and maintain adaptive organizational skills. Practitioners should assist parents in using effective parenting strategies to avoid reliance on spanking. At the same time, given the role of family socioeconomic and emotional contexts in constraining the capacity for positive parenting (Azad et al., 2014), policies aimed at reducing family poverty and alleviating parenting stress would also be beneficial for promoting children’s ATL.

### Strengths, Limitations, and Future Directions

A few limitations of this study should be noted. Notwithstanding the strong EB matching design that approximates causal estimates, the research design is still not experimental in nature given that children cannot be randomly assigned to experience spanking. Forty-four covariates were included in the matching and regression analyses, although there may still be unobserved covariates that are associated with both parental spanking and children’s ATL. Nonetheless, this study not only controlled children’s initial levels of ATL, externalizing behaviors, and executive functioning skills, but also primary caregivers’ engagement in education and home environment factors. The inclusion of the confounders and covariates is substantial progress compared to previous research in which all or some of these factors were overlooked. Moreover, the robustness of the findings excluding cases of more frequent spanking also mitigates confounding concerns about conflating frequent with infrequent spanking. The current study benefits from the longitudinal design of the ECLS-K: 2011, but because that cohort began in 2011, future research should consider similar new rigorous analyses with the ongoing ECLS-K: 2024 cohort when that data become available.

Other limitations are evident related to the spanking measure. Critically, parental use of spanking was self-reported, which is prone to social desirability bias and thereby compromises the accuracy of the measure. Some primary caregivers might minimize their report of spanking to avoid judgment given that spanking has become increasingly scrutinized, suggesting future research explore more indirect assessment approaches (e.g., Rodriguez & Silvia, 2022). Indeed, such underreporting of spanking could reduce the observed effects. Moreover, the severity of spanking could not be considered without additional data, despite measuring the frequency (i.e., 2 times or more) of spanking. For instance, hitting children “over and over as hard as one could” can be an indicator of high severity (S. Wolf & Suntheimer, 2020, p. 4). The body part where children are hit (e.g., head, back), and whether an instrument (e.g., slippers, stick, belt) or other aggressive methods of punishment (e.g., kicking, shaking, forcing children to stay in uncomfortable positions) were involved should be considered in future research. Other forms of punishment concurrent to spanking were also not available in the dataset. Future researchers should consider collecting the type, severity, and context of physical punishment delivery. For example, in the emotional content where spanking may arise, unknown interaction effects may exacerbate the potential harms of spanking, particularly considering that parental anger can intensify aggressive discipline (Rodriguez, 2018). Moreover, children’s perception of and reaction to their caregiver’s use of corporal punishment (Buck et al., 2007) could also play an unknown role in how they engage with their learning environment. Future research should consider the emotional and cognitive states of parents and children associated with spanking episodes to determine how those may mitigate or worsen the effects of spanking in educational environments.

In addition, because only biological primary caregivers’ use of spanking was analyzed in the ECLS-K: 2011, the estimate of spanking effects in this study might have been underestimated. Despite focusing on child outcomes other than ATL, previous studies have suggested that maternal and paternal use of spanking may be differentially associated with child outcomes (S. J. Lee et al., 2013; Sudo et al., 2023), such as maternal spanking being linked to behavioral problems whereas paternal spanking being linked to lower receptive vocabulary (MacKenzie et al., 2013). However, because nearly 99% of the primary caregivers were mothers, the findings of this study will be largely generalizable to the link between maternal use of spanking and children’s ATL, suggesting that future work considers the connection between paternal spanking and children’s ATL. Indeed, children who were classified in the non-spanked group may have experienced paternal spanking, potentially underestimating the cumulative effects of spanking in the home on their ATL.

Finally, the ATL measure should be interpreted with some caution. Focused on learning-related emotions and behaviors, ATL was evaluated by teachers. Although teachers’ reports were chosen over parents’ reports in order to reduce shared source variance concerns in the research design, teachers’ evaluations of non-cognitive skills like ATL can be subject to class, gender, and racial biases, and its specific appraisal requires a detailed, intersectional lens (Zimmermann & Kao, 2019). Even though children’s gender, primary caregivers’ race/ethnicity, and family poverty status were included in the matching and regression analyses, children from racial, gender, or economic minority families might have been penalized by teachers’ biases, influencing the estimates of the effects of spanking. Future researchers need to carefully interpret the complex relationship between children’s ATL skills and social stratification while monitoring the impact of biases.

## 5. Conclusions

This study found that parental use of spanking may inhibit children’s adaptive development of ATL, even if used infrequently. Banning corporal punishment in home settings would be an important step. However, solely resorting to a legal measure prohibiting corporal punishment may not be effective without public education efforts to raise awareness about the harms of spanking, parenting programs to promote positive discipline strategies, as well as community support to protect families from financial and emotional stress (Havighurst et al., 2023). Any meaningful changes to protect children from violence and promote healthy development would begin with an awareness of children’s rights and societal efforts to cultivate a safe and thriving environment for future generations.

## Figures and Tables

**Table 1:** Group Differences based on Exposure to Spanking at Age 5.5, before and after Matching.

	Before EB-Matching		After EB-Matching	
	Spanked %/M(SD)	Comparison %/M(SD)	Spanked %/M(SD)	Comparison %/M(SD)
*Child characteristics at age 5*				
Approaches to learning	2.82 *** (0.79)	3.00 (0.76)	2.84 (0.73)	2.84 (1.15)
Inhibitory control	4.62 *** (1.55)	5.02 (1.43)	4.67 (1.38)	4.67 (2.18)
Cognitive flexibility	13.93 *** (3.88)	14.43 (3.56)	13.96 (3.57)	13.96 (5.60)
Working memory	428.61 *** (33.84)	435.81 (33.88)	428.81 (32.20)	428.82 (48.64)
Externalizing behaviors	1.73 *** (0.77)	1.55 (0.71)	1.72 (0.73)	1.72 (1.27)
Boy (ref. Girls)	56 ***	0.51	0.56	0.56
Age (months)	67.57 (5.43)	67.44 (4.95)	67.50 (4.86)	67.49 (7.71)
With disability	23 ***	19	21	21
Had center-based care	54	56	53	53
Private school (ref. public)	0.12 ***	0.08	0.10	0.10
*Primary caregiver demo-graphics & behaviors*				
White (ref)	50 ***	61	47	47
Black	19 ***	10	18	18
Hispanic	23 *	21	24	24
Asian	4 *	5	7	7
Other	4 *	2	3	3
Less than HS (ref.)	11 ***	8	12	12
High school	30 ***	24	29	29
Some college	30 ***	26	29	29
Bachelor’s or higher	29 ***	43	30	30
Married (ref.)	66 ***	75	67	67
Previously married	13	11	13	13
Never married	21 ***	14	20	20
Age	32.36 *** (6.70)	34.16 (6.67)	32.49 (6.00)	32.49 (8.49)
Depressive symptoms	1.44 *** (0.91)	1.23 (0.63)	1.41 (0.74)	1.41 (1.37)
Educational expectation	76 ***	83	78	78
Volunteer for school	51 ***	60	50	50
PTO attendance	38	36	39	39
*Home environment*				
Cognitive stimulation	25.71 *** (5.00)	26.44 (4.79)	25.67 (4.90)	25.67 (8.29)
Emotional support	14.74 *** (1.83)	15.09 (1.61)	14.73 (1.65)	14.73 (2.71)
PCG Parenting stress	8.65 *** (3.10)	7.61 (2.75)	8.60 (2.76)	8.59 (4.67)
Family below poverty (ref.)	35 ***	23	34	34
Family poverty 100–199%	26 ***	21	26	26
Family poverty above 200%	39 ***	56	40	40
Discussing Religion and Family Traditions	3.75 *** (1.48)	3.63 (1.45)	3.75 (1.35)	3.75 (1.97)
Northeast (ref.)	10 ***	18	11	11
Midwest	15 ***	23	15	15
South	53 ***	34	50	50
West	22 *	25	24	24
City (ref.)	32	31	34	34
Suburb	30 ***	36	33	33
Town/rural	38 ***	33	33	33
% Covariates differed	86%		0%	
Ns ^a^	1830–1870	9340–9380	2070–2110	10,680–10,720

* *p* < 0.05, ** *p* < 0.01, *** *p* < 0.001. ^a^ Sample sizes vary across imputed data and were rounded to the nearest 10, per NCES confidentiality requirements. Source: U.S. Department of Education, National Center for Education Statistics, ECLS-K: 2011.

**Table 2:** Standardized Coefficient Results of LDV Regression Analyses for ATL Outcome, Unmatched and EB-Matched Samples.

	Spanked vs. Not Spanked	
	Age 6.5	Age 7.5
*Before matching: Full sample*		
Spanking at age 5.5	−0.079 **	−0.071 *
Caregiver parenting stress	−0.016 †	−0.033 **
(*ref. Family below the poverty line*)		
Family 100–199% of the poverty line	0.058 †	0.057 †
Family 200% above the poverty line	0.114 ***	0.097 **
*After EB-matching: Full sample*		
Spanking at age 5.5	−0.081 ***	−0.067 **
Caregiver parenting stress	−0.010	−0.043 ***
(*ref. Family below the poverty line*)		
Family 100–199% of the poverty line	0.062 †	0.106 **
Family 200% above the poverty line	0.077 *	0.125 **
*After matching: excluding spanking 2+ times (0* vs. *1)*		
Spanking at age 5.5	−0.071 *	−0.070 *
Caregiver parenting stress	−0.015	−0.042 **
*(ref. Family below the poverty line)*		
Family 100–199% of the poverty line	0.055	0.089 *
Family 200% above the poverty line	0.073 †	0.123 **

† *p* < 0.10; * *p* < 0.05, ** *p* < 0.01, *** *p* < 0.001. *Note*. Statistically significant parenting and home environment covariates were selected for reporting. Source: U.S. Department of Education, National Center for Education Statistics, ECLS-K: 2011.

## Data Availability

This study used restricted-use data, but public-use data files and user manuals are available online at https://nces.ed.gov/ecls/dataproducts.asp (accessed on 1 May 2025).

## Appendix A

**Table A1:** Standardized Coefficient Results of LDV Regression Analyses for ATL Outcome, Unmatched and Matched Samples with All Covariates Reported.

	Before Matching		After Matching	
	Spanked vs. Not Spanked		Spanked vs. Not Spanked	
	Age 6.5	Age 7.5	Age 6.5	Age 7.5
***Spanking at age 5.5***	−0.079 **	−0.071 *	−0.081 ***	−0.067 **
***Child characteristics***				
ATL at age 5	0.228 ***	0.191 ***	0.241 ***	0.193 ***
Inhibitory control at age 5	0.150 ***	0.149 ***	0.146 ***	0.152 ***
Cognitive flexibility at age 5	0.142 ***	0.106 ***	0.148 ***	0.095 ***
Working memory at age 5	0.049 ***	0.044 ***	0.043 ***	0.050 ***
Externalizing Behavior at age 5	−0.095 ***	−0.098 ***	−0.077 ***	−0.080 ***
Boy (*ref. girls*)	−0.243 ***	−0.296 ***	−0.243 ***	−0.282 ***
Age (months)	0.000	0.000	0.000	−0.004
With disability	−0.109 ***	−0.096 ***	−0.122 ***	−0.113 ***
Had center-based care	−0.004	−0.014	−0.003	−0.025
Private school (ref. public school)	−0.098 **	−0.100 **	−0.056	−0.031
***Primary Caregiver Characteristics***				
Black (*ref. White*)	−0.042	−0.057	−0.035	−0.102 *
Hispanic	0.133 ***	0.138 ***	0.157 ***	0.131 **
Asian	0.158 ***	0.250 ***	0.166 **	0.252 ***
Other	0.032	0.121 †	0.059	0.100
High school (*ref. less than HS*)	0.008	−0.001	0.030	0.015
Some college	−0.022	0.003	−0.005	0.016
Bachelor’s or higher	0.088 *	0.139 **	0.113 *	0.135 *
Previously married (ref. Married)	−0.088 **	−0.054	−0.078 †	−0.049
Never married	−0.088 **	−0.102 **	−0.080 *	−0.066 †
Age	−0.002	0.002	−0.004	0.000
Depressive symptoms	−0.012	0.002 †	−0.024 *	−0.020
Educational expectation	0.072 **	0.103 ***	0.087 *	0.082 *
Volunteer for school	0.013	0.048 *	0.031	0.029
PTO attendance	0.002	−0.007	0.020	0.014
***Home environment***				
Cognitive stimulation	0.001	−0.016	0.000	−0.018
Emotional support	−0.014	−0.009	−0.016	−0.001
Caregiver parenting stress	−0.016 †	−0.033 **	−0.010	−0.043 ***
(*ref. Family below poverty line*) Family poverty 100–199%	0.058 †	0.057 †	0.062 †	0.106 **
Family poverty above 200%	0.114 ***	0.097 **	0.077 *	0.125 **
Discussing Religion & Traditions	0.016 *	0.021 †	0.008	0.017
Midwest (*ref. Northeast*)	−0.030	−0.047	−0.032	0.016
South	0.007	−0.001	−0.010	0.040
West	−0.043	0.016	−0.029	0.015
Suburb (*ref. city*)	−0.018	0.040	−0.019	−0.045
Town/rural	−0.007	0.015	−0.020	0.023

† *p* < 0.10, * *p* < 0.05, ** *p* < 0.01, *** *p* < 0.001. Source: U.S. Department of Education, National Center for Education Statistics, ECLS-K: 2011.
